# Preoperative and early postoperative quality of life after major surgery - a prospective observational study

**DOI:** 10.1186/s12955-014-0194-0

**Published:** 2015-02-04

**Authors:** Julien Maillard, Nadia Elia, Chiara S Haller, Cécile Delhumeau, Bernhard Walder

**Affiliations:** Division of Anesthesiology, University Hospitals of Geneva, Rue Gabrielle-Perret-Gentil 4 – 1206, Geneva, Switzerland; Institute of Social and Preventive Medicine, Medical Faculty, University of Geneva, Chemin de la Tour de Champel 17 – 1206, Geneva, Switzerland; Department of Psychiatry, Beth Israel Deaconess Medical Center and Massachusetts Mental Health Center, Harvard Medical School, 330 Brookline Avenue, Boston, 02215 MA USA; Department of Psychology, Harvard University, Cambridge, 02138 MA USA

**Keywords:** SF-12, Perioperative medicine, Complications, Co-morbidity, Comfort

## Abstract

**Background:**

Changes in health-related quality of life (HRQoL) several days after surgery have rarely been investigated. We aimed to estimate the perioperative change of HRQoL, to identify patients with clinically relevant decrease in postoperative HRQoL and to establish factors associated with this decrease in HRQoL at day 30 after major surgery.

**Methods:**

Patients scheduled for major surgery at a university hospital were enrolled. Based on the HRQoL SF-12 questionnaire, the preoperative physical component summary (PCS) score, preoperative mental component summary (MCS) score, and postoperative PCS and MCS scores at day 30 were recorded. Minimal clinically important difference (MCID) was defined as those with a decrease of at least one half of the standard deviation (SD) of preoperative PCS or MCS scores. Differences between the groups with or without decreased HRQoL were investigated using univariate comparisons. A multiple logistic regression model was performed to evaluate the predictive value of potential perioperative variables.

**Results:**

The mean ± SD preoperative PCS score was 38.5 ± 10.6, postoperative score was 35.1 ± 7.8 (*p* = .004) in 85 patients. Thirty-five patients (41.2%) had a clinically relevant decrease of the postoperative PCS score. A normal to high preoperative exercise metabolic capacity measured with metabolic equivalent of task (MET) (*p* = .01) was a predictor of the decrease in postoperative PCS. The mean preoperative MCS scores (*p* = .395) were 42.2 (SD 12.8) preoperative, and 43.45 (SD 12.4) postoperative, respectively.

**Conclusions:**

Major surgery decreases postoperative PCS scores of HRQoL at 30 days. A normal to high exercise capacity was a predictor of a clinically relevant decrease of postoperative PCS scores.

**Trial registration:**

07–107 (Ethical Committee NAC of Geneva University Hospitals).

## Background

Major surgery elicits a metabolic stress response and inflammation [1]. Anesthesia and postoperative pain treatment only partially alleviate this stress reaction. Stress response and inflammation is associated with a sickness behaviour including fatigue and impaired motivation [2]. The suspected mechanism is a pro-inflammatory cytokines reaction in the brain with secondary loss of appetite, sleepiness, fever, aching joints and fatigue, and thus withdrawal from normal social activities. This sickness behaviour may impair health-related quality of life (HRQoL) shortly after surgery. Furthermore, most patients scheduled for major surgery have a significant previous history of physical and psychological suffering that likewise impairs their HRQoL [3]. These patients often anticipate a rapid improvement in their HRQoL after surgery and may underestimate changes in the early postoperative period if the outcome is different than expected [4,5]. Thus, after major surgery, patients may be at risk of both developing postoperative complications, and suffering major discomfort that can negatively affect short-term postoperative HRQoL.

Early postoperative measurements of HRQoL should be part of the outcome assessment after surgery and perioperative medicine [6], even if it is not a standard in clinical care. A poor HRQoL score may reflect poor patient satisfaction with surgery [7], as well as perioperative management, and may incur high utilization of health care resources [8]. In usual clinical care, HRQoL is estimated one or two years after surgery, which is appropriate for specific operations [9-12]. Early assessment however (i.e. one to four weeks after surgery), may more accurately reflect perioperative management and in-hospital care because of 1) short in-hospital stay [13] and 2) immediate recall of the event present (i.e. hospital stay). HRQoL immediately after the surgery has been investigated much less frequently than the ultimate outcome of surgery (i.e. cured from cancer disease or osteoarthritis) [14-17]. Trajectories of HRQoL have been rarely described after surgery. In cardiac surgery early low postoperative HRQoL was associated with low postoperative HRQoL at long term [18]. Low early postoperative HRQoL may identify patients at risk of psychological disorders, in particular anxiety and depression. Early detection would thus yield early treatment and may improve long-term outcome [19,20].

Elective intervention offers the possibility of investigating HRQoL *before* and *after* surgery, and therefore changes in HRQoL over time. Some research groups [14,17] have considered changes as clinically relevant if statistical significance was observed; for this type of investigation, however, it has been recommended to use the minimal clinically important difference (MCID) of HRQoL [21,22]. This enables surgical patients to be classified as patients with or without a clinically relevant decrease in postoperative HRQoL, making results clinically more confident. This approach for the estimation of a clinically relevant change using the MCID concept has not been tested in patients with major surgery and may allow the investigation of clinically relevant predictors of this change.

The primary aim of this study was to estimate the perioperative change of HRQoL in patients undergoing major surgery before and 30 days after surgery. Secondary aims included the identification of patients with clinically relevant decrease in postoperative HRQoL using the MCID concept and of potentially predictive factors associated with this clinically relevant decrease in HRQoL at day 30 after major surgery.

## Methods

### Design of the study

This investigation was a prospective, single-centre cohort study conducted at the University Hospitals of Geneva, Switzerland, a 2100-bed primary and tertiary care teaching Hospital. This manuscript was written according to the recommendation of STROBE checklist for the reporting of cohort observational clinical studies. Patients scheduled for major elective surgery were included between 1 April 2008 and 30 September 2008.

The study was approved by the Ethical Committee of Geneva University Hospitals (number: 07–107), Geneva, Switzerland. All procedures followed were in accordance with the ethical standards of the responsible committee on human experimentation (institutional and national) and with the Helsinki Declaration of 1975, as revised in 2000. Informed consent was obtained from all patients for being included in the study.

#### Inclusion criteria

a) Cardiac valve surgery; b) primary hip replacement; c) colorectal resection; d) resection of stomach, oesophagus or pancreas; e) femoropopliteal, femorotibial, femoro-femoral or axillofemoral bypass surgery.

#### Exclusion criteria

a) Patients with all other types of surgery, b) patients younger than 18 years of age, c) mentally disabled d) with previous diagnosis of psychotic disorder, c) were incapable of reading and writing, d) were scheduled for a second surgical intervention within 30 days after inclusion in this study to avoid interference with incomplete perioperative pathways (see below) and with measures of the study from the first intervention.

#### Patient identification

Eligible patients were identified based on weekly surgical schedules, by local research collaborators before the surgical intervention.

### Perioperative pathways for elective, major surgery

After a period of suffering the patient will consult his primary care doctor. Once first investigations and surgical problem identification are performed the patient will consult a specialist surgeon establishing the final diagnosis (potentially with supplementary exams) and the indication for major surgery. In a next step an anesthesiologist will establish a perioperative risk estimation (including pre-existing co-morbidities, exercise capacity, perioperative bleeding, and postoperative inflammation) followed by a perioperative work plan that may include additional preoperative exams and postoperative care in specialized units (recovery room, post-interventional intermediate care unit, intensive care unit). The time span between the first consultation and surgical intervention can be anything from a couple of weeks to several months. Dependent on the severity of pre-existing co-morbidities, as well as intraoperative and postoperative complications, the hospital stay may vary between one to two weeks (i.e., physical stabilization) potentially followed by weeks to months in a specialized rehabilitation clinic. Long-term follow-ups will be performed by the primary care doctor. These perioperative pathways are uniform for major surgery and independent of the type of surgery.

### Measures

#### Health related quality of life (SF-12)

HRQoL was measured using the Short-Form-12 questionnaire (SF-12) [23] from which a physical component summary (PCS) and a mental component summary (MCS) were derived. An example of a typical item for PCS is “during the past 4 weeks, does your health now limits you to climbing several flight of stairs”. Example of a typical item for MCS is “how much of the time during the past 4 weeks did you felt calm and peaceful”. The rating scales range from yes-no to likert scales, and the final score of PCS and MCS is being calculated by an algorithm (QualityMetric’s SF-12v1®). Population mean scores for the healthy European population for the PCS range from 49.4 to 51.2, and for the MCS from 47.8 to 52.9 [24] (range: 0 to 100). Lower scores mean lower health related quality of life.

#### Preoperative functional impairment

Preoperative functional impairment was assessed with two instruments by the investigators:

*Independency of health care* was assessed with one question with a yes/no answer: Is the patient independent, is the patient able to look after him/herself without any care assistance? This question estimates the absence or presence of frailty of the patient. The assessment was done by the investigators.

The *Karnofsky Score* (minimal: O; maximal: 100; normal: 100) estimates the actual functional impairment [25]. For instance, a score of 90 means “able to carry on normal activity, minor signs or symptoms of disease” and a score of 60 means “requires occasional assistance, but is able to care for most of his personal need”. A high score means no functional impairment.

#### Preoperative exercise capacity

Preoperative, exercise capacity was assessed with the *Metabolic Equivalent of Task (MET)* Score. The MET score (minimal: 1; maximum: 10; normal: >7) is an estimation of patient’s exercise capacity [26]. The MET concept was validated using exercise testing and assessing aerobic capacity [27]. This concept is recommended in the guidelines for cardiovascular assessment and management [28]. One MET means that a person’s ability is limited to activities such as eating, dressing and using the bathroom. Four METs mean that a person can climb a flight of stairs, walk up a hill, or walk on leveled ground at 4 mph. Eight METs mean that a person can do heavy work around the house like scrubbing floors or moving heavy furniture. A high score means a high exercise capacity; MET < 4 is a low exercise capacity, MET between 4 and 7 is a normal exercise capacity, MET >7 is a high exercise capacity [29,30].

#### Preoperative comorbidities

Preoperative comorbidities were assessed with three instruments by the investigators:

The *Charlson Comorbidity Index* (minimal: 0, corresponding to the highest probability of survival; maximal: 37, corresponding to the lowest probability of survival, normal index: 0) is a score that predicts the 10-year mortality in patients with comorbidities (for instance, history of myocardial infarction, dementia or tumor with or without metastasis) [31]. An algorithm weights the different comorbidities.

The *ASA* (*American Association of Anesthesiology*) *Physical Status Classification Score* (minimal: 1; maximum: 5; normal 1) is a subjective assessment of patient’s overall physical health [32]. An ASA physical score of 1 refers to a normal healthy patient with no organic or physiologic disturbance. An ASA physical score of 2 refers to a patient with history of physiologic disturbance, but without a chronic disease (for instance, arterial hypertension, diabetes). An ASA physical score of 3 refers to a patient with a severe but controlled systemic disease but with no immediate danger of death (for instance, controlled heart failure, chronic renal failure). An ASA physical score of 4 refers to a patient with severe chronic diseases with a risk of in-hospital death. An ASA physical score of 5 refers to a patient with life-threatening disease, which - with or without surgery - has high risk of in-hospital death.

The *physiological part of the POSSUM System* (minimal: 12; maximum 84; normal: 12) is part of the risk prediction of surgery [33] and has 12 categories which are investigated in the preoperative period. Examples of evaluated categories are, for instance, systolic blood pressure or abnormal electrocardiogram. The score has 1, 2, 4 or 8 points for each category (i.e., additive). A high score means high probability of 30-days morbidity and mortality.

#### Severity of major surgery

The severity of major surgery was defined by the operative part of the POSSUM System (major or major +) [33]. This operative part of the POSSUM System has 6 different categories (minimal: 6; maximum 48; normal: 6); one category is the operative magnitude (minor: 1; intermediate: 2; major: 4 and major+: 8). Patients with major or major + surgery were included in this study. Examples of major surgeries are those with severe postoperative systemic inflammation such as colorectal resection or non-aortic vascular surgery (femoropopliteal bypass surgery). Examples of major + surgeries are those with severe postoperative systemic inflammation and high risk of severe hemorrhage such as gastrectomy or resection of the pancreas. Examples of other categories are blood loss or presence of malignancy. The operative part of POSSUM is part of the risk prediction of surgery [33]. It is an additive score; a high score means high probability of 30-days morbidity and mortality.

#### Intraoperative complications

Twelve potential intraoperative complications were defined for this investigation (see below).

### Definitions of intraoperative complications

- Extended duration (>30 minutes than expected duration caused by anesthesia and/or surgical complications)
- Bleeding (blood loss >500 ml)
- Major bleeding (transfusion requirement)
- Wound contamination (according to POSSUM classification)
- Systolic arterial hypotension (decrease of >50% in the pre-induction value with a duration of >10 minutes)
- Hypoxemia (SpO2 <90% with a duration of >10 minutes)
- Hyperglycemia (increase of >30% if the pre-induction value with a duration of >120 minutes)
- Hypothermia (<35.5°C / 95.9°F with a duration of >60 minutes)
- Tachycardia (>100 beats/min with a duration of >10 minutes)
- Hyperventilation (PECO2 <30 mmHg/<4 kPa with a duration of >10 minutes)
- Acidosis (pH <7.25 with a duration of >30 minutes)
- Lactic acidosis (lactate > 2 mmol/l at any time)

#### Severity of postoperative complications

The severity of postoperative complications was defined using the Clavien complication system with 5 grades [34] (assessed by the investigators). Grade 1: Any deviation from the normal postoperative course; e.g., atelectasis (pulmonary collapsing) requiring respiratory physiotherapy. Grade 2: deviation from the normal postoperative course requiring pharmacological treatment or blood transfusion; e.g., pneumonia treated with antibiotics. Grade 3: serious postoperative complication needing considerable and supplementary interventions; e.g., arrhythmia requiring pacemaker implementation. Grade 4: life-treating postoperative complication needing prolonged stay in an intensive or intermediate care unit; e.g., heart failure leading to low-output failure. Grade 5: postoperative death. For the purpose of this study the Clavien complication system was dichotomized into grade 1 (minor complications) vs. grade 2 to 5 (moderate and major complications).

#### Postoperative comfort

The postoperative comfort was estimated using the self-report question “what is your rating for your actual overall comfort” that was rated on a 10 point likert scale ranging from 0 (worst imaginable comfort) to 10 (maximum comfort).

#### Postoperative pain

The postoperative pain was estimated using the self-report question “what is your rating for your actual overall pain in resting position” that was rated on a 10 point likert scale ranging from 0 (no pain) to 10 (worst imaginable pain).

The Aubrun analgesia protocol was used with all patients during the immediate postoperative period [35]. The protocol includes a pain assessment on a 10-point likert scale every 15 minutes. A score >3 yields the administration of an intravenous opioid dose. The dose is adjusted to the body weight and age of the patient. Thereafter, on ward, the pain assessment was performed every 6 hours by ward nurses; oral or subcutaneous opioid were administrated if the score >3. All patients with epidural and peripheral nerve catheters were followed up for 3–7 days by the acute pain service and local anesthetics were adjusted if the score was >3.

#### Postoperative itinerary

Two postoperative clinical itineraries were foreseen: 1) the simple itinerary was defined as post-anesthesia care unit (PACU) or intensive care unit (ICU) stays followed by ward stay; 2) all other itineraries were regarded as complex.

### Procedure

Previously consented patients filled in the SF-12 (inclusion between day -21 and day −1 before surgery). The post-anesthesia care unit chart, the anesthesia protocol and the surgical protocol documented details of the operation on the day of surgery (day 0). Patients recorded their responses to the comfort-related questions on day 1. Postoperative data including major complications were collected on day 2. On days 6 and 13, patients completed the same comfort questionnaire as on day 2. On days 7 and 14, the same postoperative data as on day 2 were collected. On day 28, the postoperative SF-12 was sent by mail to discharged patients (if they did not return the questionnaire they were prompted by phone), and hospitalized patients completed the postoperative SF-12 in hospital.

#### Data collection

All data were collected by the research collaborators, using standardized questionnaires at fixed times. The senior investigator was responsible for the consistency and accuracy of collected data.

### Statistical analysis

Patients were classified into 2 groups: those with and those without a postoperative decrease in HRQoL using the MCID concept. The MCID is the smallest difference that patients perceived as beneficial or harmful (impaired or decreased) and that would result in a change in patient’s management [36]. The distribution-based approach was used including sample variability (distribution) and measurement precision; this approach is used if the needed confidence estimate of MCID is not validated (which is the case for SF-12). This method is based on the assumption that there is a relationship between the MCID and its variation (i.e., standard deviation [SD]) for a specific content area. For HRQoL scores MCIDs can be estimated as one half the SD at baseline of the HRQoL score (moderate effect size) based on the universality theory of Norman and co-workers [22]. For the purposes of this study, we defined that patients with clinically relevant decreased (impaired) HRQoL at day 30 were those who showed a decrease in postoperative PCS or MCS scores of more than half of the standard deviation of the distribution of the preoperative PCS score (i.e., 5.27), or MCS score (i.e., 6.36).

Sample size requirements were estimated assuming a clinically relevant mean difference between preoperative and postoperative HRQoL (PCS) score of 5 (SD 10). The sample size needed to achieve 95% power to detect such a significant difference if it existed in the population, at an alpha level of 5% using a two-sided test, was of 52 patients. Given that our sample consisted of 100 patients, we were able to fulfill the power requests and additionally account for potential losses to follow-up (estimation was about 15%), uncompleted questionnaires (estimation was about 10%) and avoidance of overfitting related to insufficient intraoperative and postoperative adverse events per risk factor (difficult to estimate) [37].

Data were expressed using descriptive statistics with mean and standard deviation (SD), median and interquartile range (IQR), or number (percentage). The difference between preoperative HRQoL (PCS or MCS) and postoperative HRQoL was computed for each patient. First, we tested our primary aim that the difference between preoperative and postoperative HRQoL was significantly different from 0, using the Student’s t-test. If no difference was observed between pre- and postoperative HRQoL with similar SD, no logistic regression model was performed (see below).

The 2 groups were compared using univariate analyses. Categorical variables were compared using the chi-square test, and continuous variables were compared using the Student’s t-test for normal distribution or the Mann–Whitney U test for non-normal distribution. All the results were adjusted for multiples tests using Bonferroni’s correction, the reference alpha threshold was 0.15%. The normality of distribution was assessed using a Kolmogorov-Smirnov test (KS test).

We performed a logistic regression to evaluate the predictors of postoperative clinically relevant decreased HRQoL. Based on clinical importance and descriptive analyses, 5 potential predictors were chosen: independence of health care (yes vs. no), preoperative functional impairment (measured with the Karnofsky Score), exercise capacity (measured with metabolic equivalent of task (MET) recorded in 3 physiological categories (>7, 4–7 versus <4)), type of surgery (cardiac, gastric colorectal, vascular versus primary hip replacement) and pain at 6 days. These potential predictors were included, first, in a univariate logistic regression. All variables with p < 0.200 in the univariate model were entered in a multivariate logistic regression.

All statistical analyses were performed using STATA Release 12^.^0 (Stata Statistical Software Release 12^.^0, Stata Corporation, College Station, USA).

## Results

Two hundred forty-nine patients were identified, 149 had to be excluded: 12 had major language problems making informed consent impossible, 78 were unavailable for the investigators at the time of inclusion, 14 required special unplanned surgical procedures, 8 were already included in other studies, 7 had psychiatric disorders, and 30 refused to participate. A total of 100 patients were included; both HRQoL questionnaires were completed by 85 of the 100 patients. These 85 patients were analysed in details; they had completeness for 98.3% of the preoperative questionnaires and 99.0% of the postoperative questionnaires.

### Demographic variables

The mean age was 66.6 ± 11.6 years, 48% were men (Table 1). Not independent of health care at home were 11 patients (12.9%) before surgery and a low preoperative exercise capacity were estimated in 18 (21.2%). The median (IQR) duration of hospital stay was 13 days (9–16) and all patients survived.

**Table 1:** **Demographic charcteristics of 85 analyzes patients (those who completed de SF-12)**

		**Analysed n=85**
**Patient data**		
Female	n (%)	44 (51.8)
Age	mean ± SD	66.6 ± 11.6
Weight	mean ± SD	73.5 ± 15.2
Education		
Education < high school	n (%)	53 (62)
*Working situation*		
Retired	n (%)	53 (62.4)
*Marital status*		
Single/Divorced/Widowed	n (%)	32 (37.7)
Married/Living with partner	n (%)	53 (62.3)
**Pre-operative assessments**		
*Functional impairment*		
Indepedency of health care: independent	n (%)	74 (87.1)
Karnofsky score	median (IQR)	90 (90-90)
*Exercise capacity*		
Metabolic equivalent of task (MET)		
>7	n (%)	16 (18.8)
4-7	n (%)	51 (60)
<4	n (%)	18 (21.2)
*Severity of comorbidity*		
Charlson comorbidity index (adjusted for age)	median (IQR)	2 (0-4)
Charlson comorbidity index >2	n (%)	41 (48.2)
ASA	median (IQR)	2 (2-3)
ASA 1	n (%)	5 (5.9)
ASA 2	n (%)	52 (61.2)
ASA 3	n (%)	26 (30.6)
ASA 4	n (%)	2 (2.3)
Physiological section of POSSUM	median (IQR)	18 (15-23)
**Surgery**		
Primary hip replacement	n (%)	42 (49.4)
Cardiac valve surgery	n (%)	15 (17.6)
Gastric, esophageal or pancreatic surgery	n (%)	6 (7.1)
Colorectal surgery	n (%)	17 (20.0)
Femoropopliteal, femorotibial bypass	n (%)	5 (5.9)
**Severity of surgery**		
Operative section of POSSUM	median (IQR)	10 (9-13)
Major	n (%)	64 (75.3)
Major +	n (%)	21 (24.7)
**Anesthesia**		
General anesthesia alone	n (%)	62 (73.0)
Epidural/spinal anesthesia alone	n (%)	7 (8.2)
Combined anesthesia	n (%)	16 (18.8)
**Timing**		
Time from admission to surgery (h)	median (IQR)	24 (24-24)
Surgery duration (h)	mean ± SD	2.6 ± 1.3
Anesthesia duration (h)	mean ± SD	4 ± 1.5
**Intraoperative complications, number of patients**	n (%)	51 (60.0)
Major bleeding needing transfusion	n (%)	10 (11.8)
Systolic arterial hypotension	n (%)	48 (56.5)
Hypothermia	n (%)	15 (17.6)
Hyperventilation	n (%)	36 (42.4)
Lactic acidosis	n (%)	6 (7.1)
**Postoperative complications (Clavien ≥ 2)**		
Day 2*	n (%) [n]	33 (39.3) [84]
Day 7*	n (%) [n]	33 (46.5) [71]
**Postoperative comfort**		
Pain at the surgical site - Numerical rating scale (NRS)		
Day 1*	median (IQR) [n]	5 (3-7) [72]
Day 6*	median (IQR) [n]	3.5 (2-5) [68]
Pain, other - Numerical rating scale (NRS)		
Day 1*	median (IQR) [n]	0 (0-5) [72]
Day 6*	median (IQR) [n]	0 (0-4) [68]
Comfort estimation - Numerical rating scale (NRS)		
Day 1*	median (IQR) [n]	6 (5-8) [71]
Day 6*	median (IQR) [n]	7 (5-9) [67]
**Clinical itinerary**		
Simple	n (%)	74 (87.1)
Complex	n (%)	11 (12.9)
Hospital stay - days	median (IQR) [n]	13 (9-16) [50]

*Data not available for all patients.

### Difference between pre- and postoperative physical components of quality of life

The mean PCS score decreased from 38.5 ± 10.6 to 35.1 ± 7.8 30 days after surgery (*p* = .004); the average difference in PCS score was −3.4 (95%CI −5.7 to −1.1) (*p* = .004).

A MCID with decreased postoperative PCS scores was observed in 35 patients (41.2%) (Table 2). High preoperative PCS scores and higher preoperative exercise capacities (measured with the MET method) were associated with decreased postoperative PCS scores using the MCID concept in the univariate analyses (Table 2).

**Table 2:** **Comparison between patients with decreased physical component summary and patients with unchanged or increased physical component summary at day 30 after surgery (univariate analysis)**

**Physical component summary**		**Decreased n=35**	**Unchanged/increased n=50**	***p Value***
**HRQoL**				
Preoperative physical component summary (PCS)	mean ± SD	46.4 ± 9.8	33 ± 7.1	< 0.001**
Postoperative physical component summary (PCS)	mean ± SD	32.9 ± 7.9	36.6 ± 7.5	0.034
Preoperative mental component summary (MCS)	mean ± SD	41 ± 13	43.1 ± 12.8	0.461
Postoperative mental component summary (MCS)	mean ± SD	42.5 ± 12.3	44.2 ± 12.6	0.538
**Patient data**				
Female	n (%)	19 (54.3)	25 (50)	0.826
Age	mean ± SD	65.3 ± 10.7	67.6 ± 12.2	0.249
Weight	mean ± SD	73.5 ± 16.1	73.5 ± 14.7	0.980
**Preoperative assessments**				
*Functional impairment*				
Independency of health care - independent	n (%)	34 (97.1)	41 (82)	0.042
Karnofsky score	median (IQR)	90 (90-90)	80 (80-90)	0.003
*Exercise capacity*				
Metabolic equivalent of task (MET) >7	n (%)	10 (28.6)	6 (12)	0.0014**
Metabolic equivalent of task (MET) 4-7	n (%)	24 (68.6)	27 (54.0)	
Metabolic equivalent of task (MET) <4	n (%)	1 (2.86)	17 (34.0)	
*Severity of comorbidity*				
Charlson comorbidity index (adjusted for age)	median (IQR)	1.5 (0-4)	3 (0-5)	0.372
ASA	median (IQR)	2 (2-3)	2 (2-3)	0.997
Physiological part of POSSUM	median (IQR)	16 (15-23)	18.5 (15-23)	0.778
**Surgery**				
Primary hip replacement	n (%)	12 (34.3)	30 (60)	0.038
Cardiac valve surgery	n (%)	7(20)	8 (16)	
Gastric, esophageal or pancreatic surgery	n (%)	3 (8.6)	3 (6)	
Colorectal surgery	n (%)	12 (34.3)	5 (10)	
Femoropopliteal, femorotibial bypass	n (%)	1 (2.9)	4 (8)	
**Severity of surgery**				
Operative part of POSSUM	median (IQR)	10 (9-13)	10 (9-12)	0.501
Major	n (%)	25 (71)	39 (78)	0.489
Major +	n (%)	10 (29)	11 (22)	
**Anesthesia**				
General anesthesia	n (%)	25 (71.4)	37 (74)	0.165
Epidural/Spinal anesthesia	n (%)	1 (2.9)	6 (12)	
Combined anesthesia	n (%)	9 (25.7)	7 (14)	
**Timing**				
Time from admission to surgery (h)	mean ± SD	106.6 ± 290.3	44.2 ± 57.2	0.283
Time from admission to surgery; >24h	n (%)	11 (31.4)	9 (18)	0.058
Surgery duration (h)	mean ± SD	3.0 ± 1.8	2.4 ± 0.8	0.242
Anesthesia duration (h)	mean ± SD	4.3 ± 2.0	3.7 ± 1.0	0.330
**Intraoperative complications**	n (%)	21 (60)	30 (60)	1
**Postoperative complications (Clavien ≥ 2)**				
Day 2*	n (%) [n]	14 (40) [34]	18 (36) [50]	0.654
Day 7*	n (%) [n]	18 (51) [30]	19 (38) [41]	0.337
**Postoperative comfort**				
Pain at the surgical site - Numerical rating scale (NRS)				
Day 1*	median (IQR) [n]	5 (2-7) [27]	5 (3-7) [45]	0.367
Day 6*	median (IQR) [n]	3 (0-5) [28]	4 (2-5) [40]	0.074
Pain, other - Numerical rating scale (NRS)				
Day 1*	median (IQR) [n]	0 (0-5) [27]	0 (0-5) [45]	0.738
Day 6*	median (IQR) [n]	0.5 (0-5) [28]	0 (0-4) [40]	0.509
Comfort Estimation - Numerical rating scale (NRS)				
Day 1*	median (IQR) [n]	6 (5-8) [27]	6 (5-8) [44]	0.910
Day 6*	median (IQR) [n]	7 (5-8.5) [27]	8 (5.8-8) [40]	0.481
**Clinical itinerary**				
Simple	n (%)	31 (88.6)	43 (86.0)	1.000
Complex	n (%)	4 (11.4)	7 (14.0)	
Hospital stay - days	median (IQR)	14 (9-19)	12 (9-14.8)	0.580

*Data not available for all patients.

**significant result :p value < 0.0016 (Bonferroni p value threshold).

Three potential predictors were identified in the univariate logistic regression model: independence of health care, Karnofsky score, and MET (Table 3). In the multivariate logistic regression model, MET was the only independent predictor of decreased postoperative PCS scores (Table 3).

**Table 3:** **Predictors of a decrease in postoperative physical component summary (logistic regression model)**

		**Univariate model**		**Multivariate model**	
**Potential predictors**		**Crude OR (95% CI)**	***P value***	**Adjusted OR (95%CI)**	**P value**
Independance of health care	Not independent	1			
	Independent	8.5 (1.03-69.8)	0.046		
Karnofsky score		1.06 (1.01-1.10)	0.012		
Metabolic equivalent of task (MET)	<4	1		1	
	4-7	6.57 (1.37-31.6)		6.83 (1.34-34.8)	
	>7	13.3 (2.24-79.4)	0.004	11.7 (1.58-86.5)	0.012
Type of surgery	Primary hip replacement	1			
	Cardiac	2.02 (0.61-6.74)			
	Gastric	2.31 (0.41-13.0)			
	Colorectal	5.08 (1.47-17.7)			
	Vascular	0.58 (0.06-5.67)	0.073		
Pain at 6 days		0.83 (0.67-1.02)	0.082	0.81 (0.65-1.01)	0.057

OR: Odds Ratio. CI: confidence interval. P values represent the p-values of the likelihood ratio test comparing the model with and without the potential predictor.

### Difference between pre- and postoperative mental components of quality of life

The mean preoperative MCS score was 42.2 ± 12.8 and postoperative MCS score was 43.5 ± 12.4 (*p* = .395).

A MCID with decreased MCS scores was observed in 20 patients (23.5%) (Table 4). High preoperative MCS scores and higher preoperative comorbidity (measured with the ASA Physical Status Classification Score) were associated with decreased postoperative MCS scores using the MCID concept in the univariate analyses (Table 4).

**Table 4:** **Comparison between patients with decreased mental component summary and patients with unchanged or increased mental component summary at day 30 after surgery (univariate analysis)**

**Mental component summary/increased**		**Decreased n=20**	**Unchanged n=65**	***p Value***
**HRQoL**				
Preoperative physical component summary (MCS)	mean ± SD	51.2 ± 8.8	39.6 ± 12.8	< 0.001**
Postoperative physical component summary (MCS)	mean ± SD	34.6 ± 9.7	45.8 ± 12	< 0.001**
Preoperative mental component summary (PCS)	mean ± SD	39.2 ± 10	38.3 ± 10.9	0.731
Postoperative mental component summary (PCS)	mean ± SD	36.8 ± 6.1	34.5 ± 8.3	0.184
**Patient data**				
Female	n (%)	11 (55)	33 (51)	0.802
Age	mean ± SD	72.1 ± 10.7	64.9 ± 11.4	0.014
Weight	mean ± SD	73 ± 10.8	73.6 ± 16.4	0.950
**Preoperative assessments**				
Functional impairment				
Independency of health care - independent	n (%)	17 (85.0)	57 (87.7)	0.715
Karnofsky score	median (IQR)	90 (90-90)	90 (80-90)	0.882
Exercise capacity				
Metabolic equivalent of task (MET) >7	n (%)	2 (10.0)	14 (21.5)	0.338
Metabolic equivalent of task (MET) 4-7	n (%)	13 (65.0)	38 (58.5)	
Metabolic equivalent of task (MET) <4	n (%)	5 (25.0)	13 (20.0)	
Severity of comorbidity				
Charlson comorbidity index (adjusted for age)	median (IQR)	4.5 (1-5)	1 (0-4)	0.011
ASA	median (IQR)	3 (2-3)	2 (2-2)	<0 .001**
Physiological part of POSSUM	median (IQR)	23 (16-29)	17 (14-21)	0.005
**Surgery**				
Primary hip replacement	n (%)	8 (40.0)	34 (52.3)	0.039
Cardiac valve surgery	n (%)	7(35)	8 (12.3)	
Gastric, esophageal or pancreatic surgery	n (%)	0	6 (9.2)	
Colorectal surgery	n (%)	5 (25.0)	12 (18.5)	
Femoropopliteal, femorotibial bypass	n (%)	0	5 (7.7)	
**Severity of surgery**				
Operative part of POSSUM	median (IQR)	10 (9-13)	10 (9-12)	0.427
Major	n (%)	13 (65)	51 (78.5)	0.245
Major +	n (%)	7 (35)	14 (21.5)	
**Anesthesia**				
General anesthesia	n (%)	18 (90.0)	60 (92.3)	0.622
Epidural/Spinal anesthesia	n (%)	7 (35.0)	16 (24.6)	
Combined anesthesia	n (%)	5 (25.0)	11 (16.9)	
**Timing**				
Time from admission to surgery (h)	mean ± SD	73.8 (94.9)	68.2 ± 212.5	0.287
Time from admission to surgery; >24h	n (%)	7 (35)	12 (18.5)	0.251
Surgery duration (h)	mean ± SD	2.3 ± 0.5	2.7 ± 1.5	0.709
Anesthesia duration (h)	mean ± SD	3.9 ± 1.0	4.0 ± 1.7	0.452
**Intraoperative complications**	n (%)	13 (65)	38 (58.5)	0.795
**Postoperative complications (Clavien ≥ 2)**				
Day 2*	n (%) [n]	10 (50) [20]	23 (35.9) [64]	0.301
Day 7*	n (%) [n]	8 (44) [20]	25 (47) [53]	0.610
**Postoperative comfort**				
Pain at the surgical site - Numerical rating scale (NRS)				
Day 1*	median (IQR) [n]	5 (5-7) [15]	5 (3-7) [57]	0.142
Day 6*	median (IQR) [n]	2 (1-3) [16]	4 (2-5) [52]	0.032
Pain, other - Numerical rating scale (NRS)				
Day 1*	median (IQR) [n]	0 (0-5) [15]	0 (0-5) [57]	0.983
Day 6*	median (IQR) [n]	0.5 (0-3) [16]	0 (0-4) [52]	0.403
Comfort Estimation - Numerical rating scale (NRS)				
Day 1*	median (IQR) [n]	5.5 (4.6-8) [14]	7 (5-8) [57]	0.318
Day 6*	median (IQR) [n]	7 (5-8) [15]	7.5 (6-8) [52]	0.289
**Clinical itinerary**				
Simple	n (%)	16 (80)	58 (89.2)	0.278
Complex	n (%)	4 (20)	7 (10.8)	
Hospital stay - days	median (IQR)	13.5 (10-19)	12 (9-15)	0.727

*Data not available for all patients.

**significant result : p value < 0.0016 (Bonferroni p value threshold).

In absence of any differences between mean pre- and postoperative MCS score and similar standard deviation no logistic regression model was performed.

## Discussion

In this investigation we observed, first, a statistically significant postoperative decrease of HRQoL, essentially in the PCS of HRQoL 30 days after surgery. Second, we observed a clinically relevant decrease of postoperative PCS scores in 41.2% of the patients using the MCID concept. Third, the preoperative exercise capacity measured with MET scores was the only independent predictor of the clinically relevant decrease of postoperative PCS scores; higher preoperative exercise capacity was associated with decreased postoperative PCS scores using the MCID concept. Forth, no statistically significant difference between the pre- and postoperative MCS of HRQoL was observed; a clinically relevant decrease of postoperative MCS scores was observed in 23.5% of the patients.

These results are partially in line with similar investigations based on statistical significance. In a cohort of 76 patients, Aljabri and collaborators detected a statistically significant decrease in PCS (measured with SF-36) five weeks after open abdominal aortic surgery (N = 76) [14]. A statistically significant decrease of PCS at one month was also observed after gastric and colorectal surgery using the SF-12 [16,38] in cohorts of 151 and 223 patients, and a similar pattern was reported for 19 patients with liver resection surgery [39]. This statistically significant decrease of physical quality of life at one month may be related to an ongoing healing processes consecutive to an postoperative acute systemic inflammatory response syndrome after major surgery including the sickness behaviour [2], or to incomplete postoperative rehabilitation [40]. However, none of these studies analysed their data using the MCID concept, which offer more clinical confidence to results in a setting with subjective measurements and allows more in depth analyses using multivariate methods.

We observed that normal to high preoperative exercise capacity (higher MET scores) predicted decreased postoperative PCS scores using the MCID concept. A similar observation was reported after coronary artery bypass graft surgery in patients without angina [41], as well as in patients with short-stay abdominal surgery [42]. In the above-mentioned studies HRQoL was measured 3 to 4 weeks after surgery. An explanatory hypothesis could be that the perception of persisting local inflammation in the operated body part at 30 days is more intensive in preoperative healthier patients. Another hypothesis may be that healthier patients had less time to develop strategies to cope with this new situation compared to patients with preexisting exercise limitations. The observed association could be a “disability paradox” of patients with considerable burden: It is not necessary to be in good, functional health postoperatively to have higher PCS score postoperatively compared to healthier patients. In future studies these hypothetic mechanisms should be investigated because they could be treated.

The association between higher preoperative exercise capacity and clinically relevant decreased postoperative HRQoL 30 days after surgery is new and is of high clinical interest. It may change the clinical information practice and psychological preparation during preoperative consultations. To avoid exaggerated expectations of postoperative healing, this result highlights the importance to inform healthy patients that they may perceive a relevant decrease of their PCS of HRQoL in the postoperative period at least up to 30 days.

The postoperative MCS in our patients did not differ significantly from baseline and only 23.5% of the patients had a clinically relevant decrease of postoperative MCS scores. This complies with findings of earlier studies [16,38,39]. When measured before surgery and 30 days after surgery, no effects on mental health were apparent in our major surgery patients. This observation is of interest, because during this period mental health may be impaired due to transient delirium and cognitive postoperative dysfunction frequently observed after major surgery [43]. However, these adverse events seem not to have an impact on mental component of the HRQoL in most patients.

There was no difference in perioperative complications between patients with a decreased postoperative PCS and patients with an unchanged or increased postoperative PCS. However, since this study was not sampled to address this issue, our results should be interpreted cautiously. Most complications in our patients were minor and therefore probably had no impact on postoperative PCS. This was also reported in relation to adult deformity surgery [44]. Significant associations between major perioperative complications and impaired HRQoL have been reported [44].

One of the strengths of this longitudinal, prospective cohort study was the inclusion of pre-, intra- and postoperative data. Furthermore, the application of the MCID concept (instead of a statistical difference only) allowed to test associations with greater clinical relevance [22]. Last but not least, previously validated measurement instruments for preoperative and postoperative analysis of HRQoL [25,31,33] were used.

The relatively small sample size limits statistical power, thus observed differences between groups (decreased HRQoL vs. stable HRQoL) should be interpreted with caution, and demands further validation in larger samples. The concept of MCID in this study was only used for decreased HRQoL and not for improved HRQoL based on the valid evidence that HRQoL is decreased after major surgery in the first month [14,16,38,39].

## Conclusion

Major surgery significantly decreased postoperative PCS of HRQoL at 30 days, but not MCS. More the 40% had a clinically relevant decrease of PCS scores and more than 20% a decrease of MCS scores. Normal to high preoperative exercise capacity was predictive for a clinical relevant decrease of postoperative PCS.

## Abbreviations

ASA: American association of anesthesiology
HRQoL: Health-related quality of life
ICU: Intensive care unit
IQR: Interquartile range
MCID: Minimal clinically important difference
MCS: Mental component summary
MET: Metabolic equivalent of task
NRS: Numerical rating scale
PACU: Post-anesthesia care unit
PCS: Physical component summary
SD: Standard deviation
SF-12: Short-form 12
